# Food Authentication: Truffle (*Tuber* spp.) Species Differentiation by FT-NIR and Chemometrics

**DOI:** 10.3390/foods9070922

**Published:** 2020-07-13

**Authors:** Torben Segelke, Stefanie Schelm, Christian Ahlers, Markus Fischer

**Affiliations:** Hamburg School of Food Science—Institute of Food Chemistry, University of Hamburg, Grindelallee 117, 20146 Hamburg, Germany; torben.segelke@chemie.uni-hamburg.de (T.S.); stefanie.schelm@chemie.uni-hamburg.de (S.S.); christian.ahlers-2@studium.uni-hamburg.de (C.A.)

**Keywords:** truffle, *Tuber* spp., food authentication, species differentiation, near-infrared spectroscopy, chemometrics

## Abstract

Truffles are certainly the most expensive mushrooms; the price depends primarily on the species and secondly on the origin. Because of the price differences for the truffle species, food fraud is likely to occur, and the visual differentiation is difficult within the group of white and within the group of black truffles. Thus, the aim of this study was to develop a reliable method for the authentication of five commercially relevant truffle species via Fourier transform near-infrared (FT-NIR) spectroscopy as an easy to handle approach combined with chemometrics. NIR-data from 75 freeze-dried fruiting bodies were recorded. Various spectra pre-processing techniques and classification methods were compared and validated using nested cross-validation. For the white truffle species, the most expensive *Tuber magnatum* could be differentiated with an accuracy of 100% from *Tuber borchii*. Regarding the black truffle species, the relatively expensive *Tuber melanosporum* could be distinguished from *Tuber aestivum* and the Chinese truffles with an accuracy of 99%. Since the most expensive Italian *Tuber magnatum* is highly prone to fraud, the origin was investigated and Italian *T. magnatum* truffles could be differentiated from non-Italian *T. magnatum* truffles by 83%. Our results demonstrate the potential of FT-NIR spectroscopy for the authentication of truffle species.

## 1. Introduction

Today’s globalization leads to an increase of known cases of food fraud [1]. At the same time, consumer demand is moving towards food products of higher quality [2]. Many cases of food fraud pose a risk to health if toxic or allergenic substances get into the products through adulteration. However, even in cases of food fraud, which in many cases do not lead to a health hazard, it must be ensured that the consumer is not economically harmed, i.e., that no unjustifiably high prices are charged for inferior goods.

The increasing interest of the consumer in higher quality food [3], and also the willingness to pay more money for it, provides the incentive for criminally motivated actors to stretch high-end products with cheaper ingredients. Since many falsifications cannot be detected immediately by laymen or even by trained personnel in companies, it is becoming increasingly important to have appropriate instrumental detection methods for possible food adulteration at hand [4].

Because of the unique aroma and taste emitted from the fruiting bodies, truffles (*Tuber* spp.) are considered as delicacies. The underground growing ascomycetes represent the most expensive of all edible fungi, whereby the white Piedmont Truffle (*Tuber magnatum*) and the black Périgord Truffle (*T. melanosporum*) are the most valuable species: prices do range between 3000–5000 €/kg and 700–1200 €/kg, respectively [5,6,7].

Because of their high price, truffles are often subject to fraud, especially when the species are very similar in their morphological appearance: *T. borchii* (syn. *Tuber albidum* Pico) is a truffle morphologically and biochemically similar to *T. magnatum*, both are classified as white truffles. The latter is the most expensive truffle species of all, so it is obvious that it is the subject of an intended counterfeit [8,9]. However, even unintentional cases of fraud are reported when other truffles, such as *T. borchii* are harvested, although the roots have initially been colonized by *T. magnatum* [10,11].

Amongst black truffles, the species *T. melanosporum* is the most expensive and highly valued for its organoleptic properties [12]. The Asian black truffles (e.g., *Tuber indicum*, *Tuber himalayense,* and *Tuber sinense*) form fruiting bodies morphologically very similar to *T. melanosporum* [13]. In view of the higher price of *T. melanosporum*, there is also a risk of fraud, especially since Asian black truffles are imported into Europe from China [14,15,16].

Due to the above-mentioned potential fraud cases, analytical authentication techniques are necessary, which must also be time-efficient due to the short-term storage of the industry.

In 2006, Zhao et al. compared five Chinese truffle fruiting bodies using Fourier transform infrared (FT-IR) spectroscopy [17] and successfully differentiated *T. magnatum*, *T. indicum,* and *Tuber excavatum* from each other. More recently, El Karkouri et al. proposed a matrix-assisted laser desorption/ionisation time of flight mass spectrometry (MALDI-TOF-MS) strategy, analysing proteins and applying database search algorithms [5]. In 2020, Krauß et al. analysed different tuber species regarding their geographical origin and species authentication via stable isotope ratio analysis showing that a differentiation with this method is possible [18]. However, these techniques still require costly instrumentation, maintenance and sophisticated handling. Instead, our practical approach is, to our knowledge, the first Fourier transform near-infrared (FT-NIR) spectroscopy study addressing the authentication of truffles with a relatively large number of samples.

FT-NIR spectroscopy is a simple and cost-effective approach, nowadays widely used for the monitoring as well as for the controlling of product quality and safety [19] alike the evaluation of the freshness [20] or of pesticide residues of fruits and vegetables [21]. FT-NIR spectroscopy is widely used for the authentication of foodstuffs [9,22,23,24] or for controlling the intentionally or unintentionally adulteration of exogenous substances or process by-products [25,26,27] and was recently used to monitor the post-harvest ripening of white truffles [28].

Data pre-processing of the obtained data is a crucial step in spectroscopic analysis. Therefore, pre-processing techniques, such as scatter correction, smoothing, or detrending steps are used in order to reduce the variability between samples due to scattering caused e.g., by heterogeneous sample size of powdery samples. Furthermore, additive and multiplicative effects in the spectra are removed and a subsequent exploratory analysis, a bi-linear calibration model or a classification model is improved [29]. It is essential to carefully compare and select the data pre-processing techniques to avoid misleading results and overfitting [29,30,31]. The decision on the classification model is crucial as well, and therefore, similarly to the evaluation of different data pre-treatment steps, we have examined and compared various classification models.

The aim of this study was to develop a reliable, easy-to-handle and low-cost method using the FT-NIR technology coupled to chemometric tools for the differentiation and authentication of five economically relevant truffle species. In this regard, we concentrated on the real truffles of the genus *Tuber* defined in the German Guidelines for mushrooms and mushroom products [32] and used in foodstuffs: the expensive species *T. melanosporum* and *T. magnatum,* as well as the less expensive species *T. aestivum*, *T. borchii,* and *T. indicum*. In this study, 75 truffle samples from three years of harvest and eleven growing countries were analysed. Different common pre-processing techniques were applied to the raw spectra and the results were compared using various classification models.

## 2. Materials and Methods

### 2.1. Sample Acquisition

In total, 75 truffle samples of relevant, market available white and black truffle species (harvest years 2017–2020) from 11 different countries were analysed in this study.

More precisely, the sample set consisted of two white species *T. magnatum* (20 samples) and *T. borchii* (5 samples) and three black species *T. melanosporum* (10 samples), *T. aestivum* (synonym *T. uncinatum* [33], 29 samples), and *T. indicum* (11 samples).

Regarding the *T. aestivum* species, molecular biological analyses have shown that *T. aestivum* and *T. uncinatum* are one species. Both terms should therefore be regarded as synonymous. Since *T. aestivum* was described before *T. uncinatum*, the species should be named *T. aestivum* [33]. Based on these molecular biological findings, *T. aestivum* and *T. uncinatum* were subsumed and named *T. aestivum* in this study.

An overview of the collected samples is given in Table S1. Some samples were commercially purchased and, therefore, considered as non-origin-authentic, so the origin is stated as ‘unknown’ in Table S1. Still, information regarding the truffle species were secured for all samples either by personal participation in harvest or by DNA analysis carried out within the Hamburg School of Food Science [34]. On arrival, all samples were frozen in liquid nitrogen and stored at −80 °C until further treatment.

### 2.2. Sample Preparation

Per sample, several fruiting bodies, at least 75 g, were cleaned with pure water obtained by a Direct-Q purifying system (Merck Millipore, Burlington, MA, USA) for removing remaining soil. Subsequently, the fruiting bodies were milled using a knife mill (Grindomix GM 300, Retsch, Haan, Germany) with dry ice at a ratio of 1:1 (*w*/*w*) and freeze-dried for 72 h [24]. The truffles were freeze-dried because of two reasons, which are more discussed in Section 3.1: (i) FT-NIR spectra of fresh truffles showed unspecific spectra with large water bands. (ii) It was known from the literature that such a freeze-drying step can enhance the accuracy of the classification models [35]. Freeze-dried material was crushed using a mortar and a pestle to obtain a fine homogeneous powder.

### 2.3. Spectra Acquisition

For the acquisition of near-infrared spectra, a TANGO FT-NIR spectrometer with an integrating sphere (Bruker Optics, Bremen, Germany) was used. The signals were recorded between 11550–3950 cm^−1^, collecting 50 scans at a resolution of 4 cm^−1^. All spectra were acquired at room temperature of 22 ± 2 °C. Samples of 300 mg, weighed in a glass vial (52.0 × 22 mm × 1.2 mm, Nipro Diagnostics Germany GmbH, Ratingen, Germany), were analysed in triplicate, in-between individual spectra recordings the lyophilisate was shaken in the glass vial.

### 2.4. Spectra Pre-Processing

FT-NIR spectra were pre-processed using MATLAB R2019a (The MathWorks Inc., Natick, MA, USA). After having omitted a specific range of higher wavenumbers (see Table 1 and discussion below), different pre-processing techniques or combinations of them were applied and compared (see Table 1) [36].

Multiplicative scatter correction (MSC) using the average of all spectra as the reference spectra was performed to eliminate scatter effects for all approaches i–vi. First order derivate (approach ii) was calculated to eliminate offset, baseline drifts and additive scattering effects, and second order derivate (approach iii) was calculated to remove multiplicative scattering effects in beyond. Detrending (polynomial order = 1) was applied for approach iv and vii. The effect of smoothing (moving average, span = 5) before MSC was investigated for approach v–vii.

After the pre-processing methods stated in Table 1, a binning by averaging 10 adjacent features was carried out with all spectra. Lastly, the triplicate spectra were averaged [24,25,36,37]. For certain issues (e.g., only black or white truffles or origin determination of *T. magnatum* samples), the MSC correction was only applied to the selected spectra.

### 2.5. Multivariate Data Analysis

For data investigation and visualization, principal component analysis (PCA) and line plots were calculated using MATLAB R2019a after applying spectra pre-treatments and mean centring the data.

For the different pre-processing approaches i–vii (see Table 1) it was each evaluated which classification model achieved the best prediction accuracy using MATLAB R2019a. The classification models examined in this context are stated in Table 2.

For optimising the model parameters and for obtaining an unbiased estimate of the model’s performance, stratified nested cross-validation was used [44,45]. Therefore, the whole data set was split into four parts whereby the samples were stratified by the species to ensure a representative and balanced training set (three fourths) and test set (one fourth). For the training set, 10-fold cross validation was applied to select the optimal model parameters, referred to as inner cross-validation. The performance of the calculated model was then evaluated by predicting the test set. This process was repeated for all four folds, so every part of the four-fold outer cross validation was once used as the test set.

Finally, since the results by a single nested cross validation can vary, the entire nested cross-validation and the prediction of the test set were repeated 100 times, of which the mean accuracy and the standard deviation are reported.

## 3. Results and Discussion

### 3.1. Spectra Investigation

**Figure 1:**
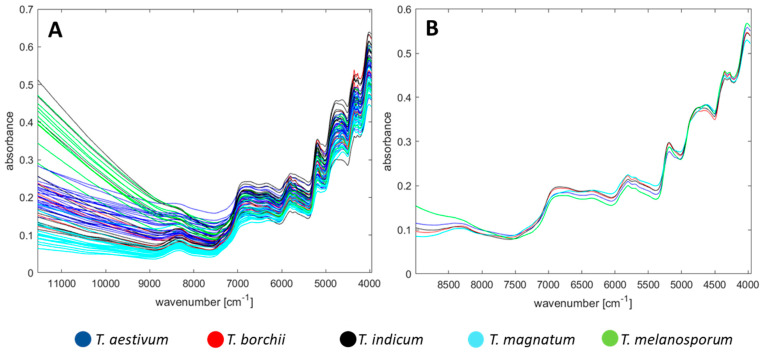
(**A**) Raw Fourier transform near-infrared (FT-NIR) spectra, triplicate measurements from all 75 samples, coloured by truffle species. (**B**) Mean FT-NIR spectra for each truffle species after omitting the >9000 cm^−1^ range, MSC and binning.

Figure 1A shows all untreated spectra of the raw data, coloured in accordance to the different truffle species. As anticipated and seen from Figure 1A, the absorbance rises towards lower wavenumbers because of the transition probability which is higher for the first transition than for higher overtones [46].

However, in the range from 11,550−9000 cm^−1^ some spectra show strong absorbance. Calculating the corresponding wavelength, this region from 11,000–9000 cm^−1^ relates to the region from 1111−909 nm, which is close to the visual region. Here, the 4^th^ overtone of the –OH bond occurs, and the colour of the truffle lyophilisate itself might cause an offset, which could have increased the absorbance [47]. Since the spectra vary in a strong way for this region, chemometric analyses, such as PCA, would excessively focus on this region and would neglect the information that is present in the spectra for smaller wavenumbers, so we excluded the >9000 cm^−1^ region. In fact, the range >9000 cm^−1^ is often excluded in various FT-NIR studies—also because this region is prone to noise when performing data pre-processing methods, such as first or second derivative [37,43].

Regarding the exclusion of some regions in the FT-NIR spectra, special care has to be taken to bands, which can be affected by the water content. Particularly in the region around 5312 cm^−1^ (O−H stretching, first overtone) and around 7142 cm^−1^ (O−H deformation, second overtone), water can affect the absorbance of protein or carbohydrate specific bands [43]. The analysis of fresh truffle samples has shown that a drying step is necessary, as otherwise large water bands and unspecific spectra are obtained which superimpose the information beneath. Thus, the truffle samples were freeze-dried because such a sample preparation can enhance the accuracy of the classification models [35]. Due to the freeze-drying step, the water content in the samples can be seen as negligibly small and in the same range, so it should have no impact on the differentiation with chemometric models in the following steps. In addition, in the region 6500−5300 cm^−1^, not only water molecules absorb electromagnetic radiation, but also C–H vibrations do, which could be a useful parameter for the differentiation of the truffle species. In order to avoid the loss of useful information, we have not excluded other regions for this non-targeted approach, as several other research groups do in practice [24,37].

For powdered samples, multiplicative scatter effects occur due to differences in the materials’ particle size, and have to be corrected for a reasonable data interpretation. To overcome such scattering effects, two approaches are commonly used: MSC and standard normal variate (SNV). According to Dhanoa et al., both pre-processing steps are two alternative approaches, which lead to similar results [48]. In the present study, MSC was chosen to correct for scattering effects. It should be noted that the sequence of the various pre-processing steps is always decisive. In Figure S1, the effect of applying MSC on the raw data, after having omitted the >9000 cm^−1^ region, is shown. On the contrary, applying MSC first and omitting the >9000 cm^−1^ region afterwards will have misleading results, as shown in Figure S1B on the right: the unwanted variance in the >9000 cm^−1^ region is not excluded, but persists in the spectra as an error propagation. By applying pre-processing steps, it is therefore important to examine and to compare the impact of different orders, noted in the same way by Gerretzen et al. [49]. Any further pre-processing steps will be investigated and discussed more deeply in Section 2.4.

### 3.2. Spectra Interpretation and Assignment of Bands

The FT-NIR spectra reflect the major constituents of the truffles. Naturally low in fat, lyophilised truffle samples are rich in dietary fibre and proteins [50]. These components can be recognised in the spectrum by their characteristic bands; however, it should be noted that an exact assignment of bands for complex samples is difficult due to overlapping effects. For the sake of clarity, the mean spectra have been calculated for each truffle species, and the resulting representation is shown in Figure 1B. At around 6667 cm^−1^ a vast band can be located induced by N−H stretching (first overtone) that can be attributed to proteins and amino acids. Furthermore, N−H combinations are also present around 4687 cm^−1^ and the bands at 4859 cm^−1^ and 4600 cm^−1^ are caused by amide groups [24,38,47].

Regarding the carbohydrates, the double peak at 4338 cm^−1^ and 4257 cm^−1^ can be assigned to −CH_2_ asymmetric stretching and symmetric stretching, respectively [51]. In addition, C−H stretching (first overtone) and −CH_2_ vibration lead to peaks at 5760 cm^−1^ and 5742 cm^−1^, respectively [24,37,47].

In order to put these observations into context, the work of Saltarelli et al. with an analysis of the protein and carbohydrate content of *T. magnatum*, *T. borchii*, *T. aestivum,* and *T. melanosporum* is of great importance. Although their work did not emphasise the species differentiation but storage effects, they have already noticed differences in the major constituents for the truffle species [52]. This can be illustrated well e.g., by the protein fraction: In ascending order, *T. melanosporum*, *T. aestivum*, *T. borchii,* and *T. magnatum* have a soluble protein content of 8.7, 11, 13, and 24%, respectively [52]. Such an order can be found at the wavenumber 6318 cm^−1^: *T. melanosporum* showing the lowest absorbance for this protein-specific region and *T. magnatum* the highest, so the above-mentioned study and our FT-NIR analysis is therefore consistent. Admittedly, this order is not properly given over the entire protein-specific range, especially *T. magnatum* shows an individual curve, but it should be noted that FT-NIR analysis is not capable of specifically measuring soluble proteins, as Saltarelli et al. (2008) did in their approach. Instead, it returns a general parameter, so the amount of scleroprotein and non-soluble protein fractions could cause the discrepancy. Consequently, it should be possible to distinguish species by—albeit very costly—quantitation of soluble protein and carbohydrate content. FT-NIR analysis, on the other hand, enables the indirect and rapid identification of these major constituents.

### 3.3. Principal Component Analysis

PCA is widely used for visualising high dimensional data by transforming them into a low dimensional space. As an unsupervised approach, it is useful for the qualitative data exploration, checking for potential outliers and rechecking the research hypothesis before using supervised classification models [53,54].

**Figure 2:**
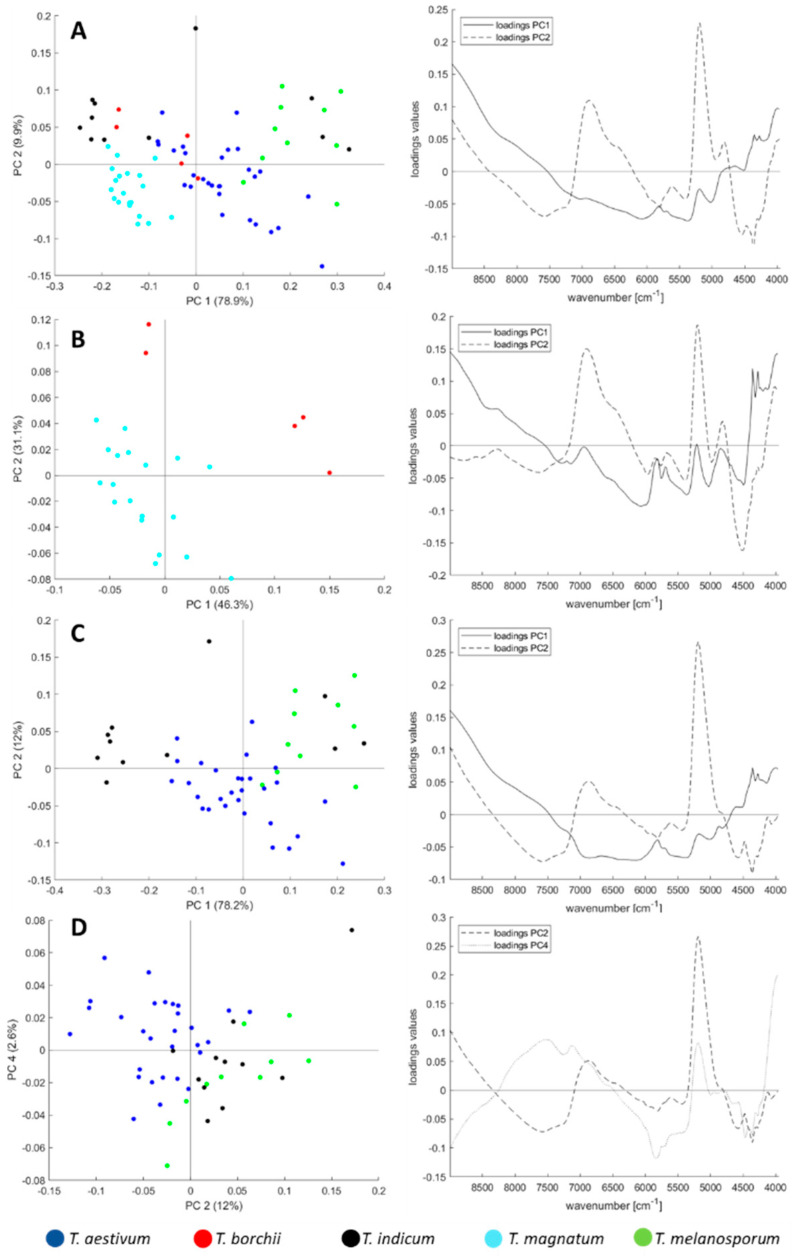
Principal component analysis (PCA) score-plots with their respective loadings plots after pre-processing approach No. i of (**A**) all five truffle species, (**B**) only white truffle species, and only black truffle species in the (**C**) PC2 vs. PC1 plane and (**D**) in the PC4 vs. PC2 plane.

Figure 2A shows the score-plot for all 75 truffle samples. Tendencies of cluster formations according to the truffle species can be identified: the *T. magnatum* samples are located in the lower-left, whilst the *T. melanosporum* samples are located to the right and the *T. aestivum* samples are in the centre of the plot. *T. borchii* und *T. indicum* samples scatter across the plot. These intermediate results give reason to assume that a classification of truffle species is possible. However, with a differentiation of all five species we do not address real issues in the incoming goods inspection: the truffle’s colour can be checked visually; thus, it only makes sense to consider the white and black truffles separately especially because falsification occurs within the white and within the black truffle, and these are not adulterated with each other. Therefore, PCA was calculated only for white and black truffle species and the score-plots are shown in Figure 2B–D, respectively. The trends from the score-plot in Figure 2A are also noticeable here, and FT-NIR analysis appears to be an appropriate method for differentiating truffle species. For the *T. indicum* samples in Figure 2C, some samples are spread over the entire score-plot, but tend to higher PC2 values in the PC4 vs. PC2 plane, already indicating the need for multivariate, non-linear analysis tools hereinafter. Moreover, the fact that there is still cluster formation shows that the important information for the species differentiation is not only contained in the >9000 cm^−1^ region, which was omitted, but is present over the whole spectra.

### 3.4. Evaluation of Pre-Processing and the Suitability for the Species Classification

Whereas applying MSC or SNV correction is necessary without question and is common practice in FT-NIR studies, the need and the impact of any further pre-processing steps should be investigated experimentally [55]. For evaluating the quality of such steps, only visual comparison of ‘before-and-after’ PCA plots is unlikely to find the most suitable pre-processing strategy and may mislead to an approach, which is not appropriate for a supervised model, so we calculated classification models and compared the prediction accuracy [36,49].

**Figure 3:**
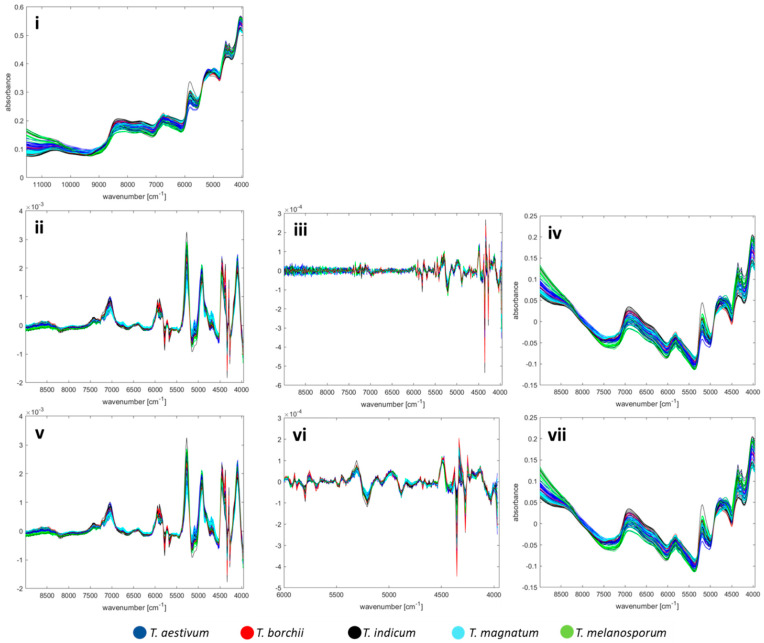
Spectra comparison of different pre-processing approaches, also refer to Table 1**.** First row: one-step pre-processing: (**i**) MSC. Second row: two-step pre-processing: (**ii**) MSC, 1st derivative. (**iii**) MSC, 2nd derivative. (**iv**) MSC, detrending. Third row: three-step pre-processing: (**v**) smoothing, MSC, 1st derivative. (**vi**) Smoothing, MSC, 2nd derivative. (**vii**) Smoothing, MSC, detrending.

Spectra comparison of different pre-processing approaches examined are shown in Figure 3. The effect of smoothing is not recognisable visually. In addition, it turned out that neighbouring wave numbers show almost identical absorbance values. In order to avoid redundant data and overfitting, a binning was carried out by calculating the mean value of the absorbance for 10 adjacent wavenumbers and combining the measuring points into 248 variables.

For every pre-processing approaches, all five classification models stated in Table 2 were calculated and validated using stratified nested cross-validation. As the main result parameter for comparing the approaches, we used the mean accuracy instead of the overall accuracy to account for the different size of the groups. The classification accuracies and precision for the test set for the differentiation of white and black truffles are given in Table 3 and Table 4, respectively. For the training set used for validation, the classification accuracies and precisions are given in Tables S2 and S3, respectively.

As can be seen in Table 3, all classification models provide good accuracy (>90%). Only the second derivation leads to significantly worse results. A pre-treatment of MSC with first derivation with both a linear and a quadratic SVM lead to an error-free classification of 100% (the most appropriate results are marked bold in the corresponding tables). Accordingly, any falsification of the expensive *T. magnatum* with the cheaper *T. borchii* can be detected. Because of the clear result based on the available and analysed truffle samples, the confusion matrix is not needed here, but can be seen in the supplement in Table S4.

A similar trend can be seen for the black truffles: Here too, high accuracies are generally achieved (>90%), only the second derivative without previous smoothing performs worse and a linear model does not seem to be sufficient for this ternary issue. Although the results overlap when the standard deviation is considered, the best accuracies of 99.1 ± 1.2 % are obtained when using MSC and the first derivative with the SSD model. A previous smoothing does not yield a significant improvement. Since every data pre-treatment is also a manipulation of the data, the model with the fewest steps should be preferred. The corresponding confusion matrix is shown in Table 5. In particular, fraud is common with *T. indicum*, which is counterfeited as the high-priced *T. melanosporum* because the two species are morphologically very similar and collected at the same harvesting times. Therefore, it is pleasing that the specificity for *T. melanosporum* is 97.5%—the error rate of mistakes is only 2.5%.

Table S5 shows the classification results for the test set for the differentiation of all five truffle species, indicating that also for this more complex five-class-issue, classification models can be calculated with high accuracy of 99%, and for the training set used for validation, the classification accuracies and precisions are given in Table S6. The corresponding confusion matrix is shown in Table S7.

DNA analysis is often used to authenticate species and varieties, while FT-NIR analysis is widely established in industrial incoming goods inspection. FT-NIR analysis does not require any specialised training for handling and any special, eventually hazardous chemicals for sample preparation and measurement, therefore the FT-NIR analysis is a “green method” [56]. Additionally, possible contamination due to exponential amplification by PCR quickly leads to false positive results. In order to keep this danger to a minimum, separate laboratories for sample preparation and DNA analysis are necessary, whereas NIR does not have such requirements. Optionally, it would be conceivable to use FT-NIR measurement for sample screening and to countercheck any conspicuous results by DNA analysis.

Regarding the determination of the geographical origin, however, DNA analysis cannot provide reasonable answers since the origin rather affects the phenotype. Here, FT-NIR analysis can be a tool for differentiating the origin [35] and the possibilities for the truffle differentiation by origin are examined in the following chapter.

### 3.5. Influence of Harvest Year and Geographical Origin

As shown in the PCA plot (Figure 2A), the truffle species has the dominant influence on the NIR spectrum, since the scores cluster according to their species in this unsupervised model. This can be demonstrated on the *T. magnatum* samples, which, although dominant from Italy, originate from Bulgaria, Croatia, and Romania, and are clustering together in the unsupervised PCA. This effect is similar for the *T. aestivum* samples originating mainly from Romania, but also from Bulgaria, France, Iran, Italy, Moldovia, and Slovenia. Thus, the species itself seems to have a much greater influence on the metabolome to be measured by FT-NIR spectroscopy than the origin.

One model for the origins of all truffle samples is not advisable for this reason, since most Italian samples are white truffles and most Romanian samples are *T. aestivum* truffles what is linked to their natural areas of origin. Such a model might, therefore, correlate on a false causality. However, the price depends primarily on the species whilst the origin is a second factor in the purchase decision. Accordingly, for the incoming goods inspection it is important especially for the most expensive *T. magnatum* truffle whether it comes from Italy or not, according to the consumer’s expectations. For this Italy vs. non-Italy issue, all pre-processing was compared with classification models, analogous to the previous investigations when targeting the species. The results of the test set are shown in Table 6, and for the training set used for validation, the classification accuracies and precisions are given in Table S8. Best classification results of 88.4 % are reached after MSC and 2nd derivative in combination with a Random Forest (RF) classification model. However, we have decided not to pursue this pre-processing strategy because the spectra line plots in Figure 3iii have shown that a lot of noise occurs in the range of wavenumbers above 6000 cm^−1^ and a smoothing an omitting this range is preferable. This alternative approach leads to a slightly worse accuracy of 82.8 ± 8.1% and the corresponding confusion matrix is shown in Table 7. The accuracy results provided by the LDA classification only differ by a few percentage points, and are even better in some cases. However, we chose the RF model since the PCA plots have arouse the impression that non-linear classification models might be more suitable for this issue.

**Figure 4:**
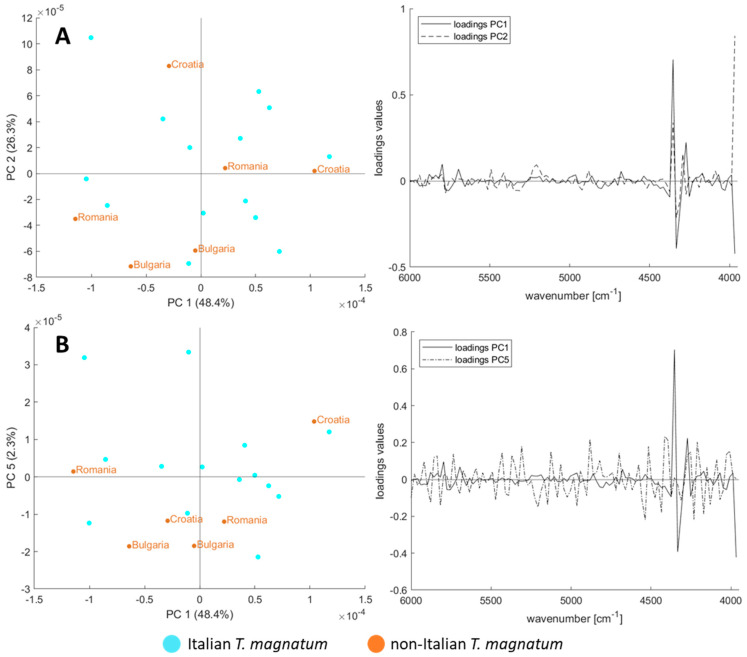
PCA score-plots with their respective loadings plots after pre-processing approach No. vi of the *T. magnatum* samples from Italy and other countries (**A**) PC2 vs. PC1, (**B**) PC5 vs. PC1.

Additionally, the PCA-plots for the *T. magnatum* samples were calculated and are shown in Figure 4, indicating and confirming that a non-linear classification model, such as RF, is more suited for this issue. Still, there are two aspects to consider: first, the standard deviation is remarkably high and second, the PCA plots show that the variance within the Italian samples is at least as large as the variance of the other origins. An origin model with acceptable accuracy is chemometrically possible, but should be checked with additional samples.

**Figure 5:**
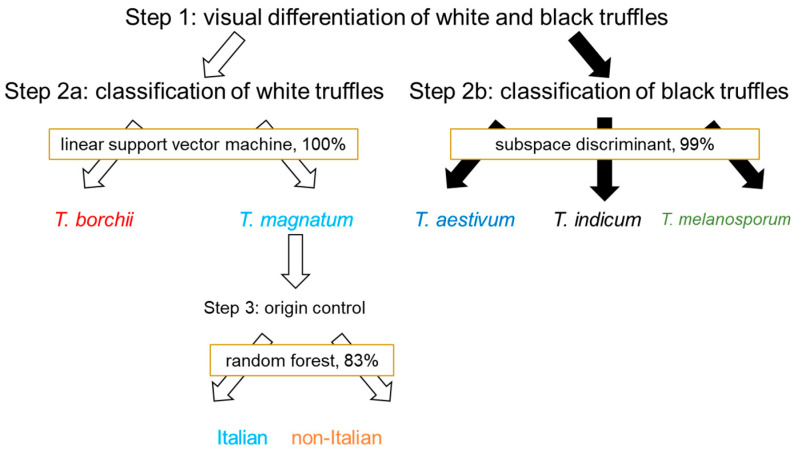
Authentication protocol for the stepwise authentication assessment of truffles with FT-NIR and chemometrics.

As the results show, FT-NIR can be used for the differentiation of black and white truffles, and Italian and non-Italian truffles of the species *T. magnatum*. Since FT-NIR is a simple and cheap method, it is suitable for industrial applications, for example, for the incoming goods inspection or authenticity checks on truffles. The process of authentication using FT-NIR is shown schematically in Figure 5.

## 4. Conclusions

FT-NIR spectroscopy was combined with chemometrics to distinguish within the white truffles *T. borchii* and *T. magnatum* and the black truffles *T. aestivum*, *T. indicum,* and *T. melanosporum*. Different techniques for pre-processing in combination with various classification models and their effect on the accuracy of the model were compared. Classification accuracies >99% showed that the analysis of truffle samples by FT-NIR spectroscopy is a very suitable tool for species differentiation without sophisticated sample preparation or instruments. When differentiating between Italian and non-Italian *T. magnatum* samples, an accuracy of 83% was achieved. FT-NIR analysis requires no special training for handling and no special, possibly hazardous chemicals for sample preparation and measurement. In addition, most quality assurance laboratories already have FT-NIR instruments. Due to its simple, cost-effective application, FT-NIR analysis is very well suited for industrial screening samples during incoming goods inspection. Considering the number of 75 truffle samples used, we intend to extend the results of our study by analysing further samples, including a research on the potential effects of the harvest year.

## Figures and Tables

**Table 1:** Pre-processing steps to the raw spectra in the order 1–2–3. For all approaches, a binning was added as a last step. MSC, multiplicative scatter correction.

Approach No.	Cut	Smoothing	MSC	1st Derivative	2nd Derivative	Detrending
**(i)**	>9000		1			
**(ii)**	>9000		1	2		
**(iii)**	>9000		1		2	
**(iv)**	>9000		1			2
**(v)**	>9000	1	2	3		
**(vi)**	>6000	1	2		3	
**(vii)**	>9000	1	2			3

**Table 2:** Overview of the classification models examined in this study.

	Classification Models	Hyperparameters Used	References
**a**	Linear Discriminant Analysis (LDA)	discrimination type: linear	[38]
**b**	Linear Support vector machine (lin. SVM)	kernel function: polynomial polynomial order = 1 kernel scale = 1 box constraint level = 1	[23,24,35,37,39]
**c**	Quadratic Support vector machine (quad. SVM)	kernel function: polynomial polynomial order = 2 kernel scale = 1 box constraint level = 1	
**d**	Subspace Discriminant (SSD)	method: subspace learners: discriminant number learning cycles = 30	[40]
**e**	Random Forest (RF)	split criterion: Gini’s diversity index max. number of splits = 100:	[41]
**f**	*k*-nearest neighbour (*k*-NN)	number of neighbours = 1 distance: Euclidean distance weight: equal	[22,42,43]

**Table 3:** Mean accuracy and precision of the prediction of the external test set for different pre-treatments and classification models for the differentiation of the white truffle species (20 *T. magnatum* samples and 5 *T. borchii* samples, all values in %).

			Classification Models				
		(a) LDA	(b) lin. SVM	(c) quad. SVM	(d) SSD	(e) RF	(f) *k*-NN
**pre-processing**	(i) MSC	99.2 ± 1.1	91.2 ± 3.9	98.8 ± 1.4	98.7 ± 1.5	98.7 ± 3.2	99.1 ± 1.2
	(ii) MSC, 1st derivative	99.9 ± 0.4	**100 ± 0.0**	100 ± 0.0	99.8 ± 0.7	94.7 ± 3.4	98.7 ± 1.4
	(iii) MSC, 2nd derivative	98.0 ± 1.7	87.1 ± 1.9	94.9 ± 2.1	97.7 ± 2.0	96.3 ± 4.2	93.8 ± 2.0
	(iv) MSC, detrend	98.9 ± 1.3	94.2 ± 3.5	99.5 ± 0.9	99.0 ± 1.4	97.9 ± 2.6	99.1 ± 1.2
	(v) smoothing, MSC, 1st derivative	99.8 ± 0.6	99.5 ± 0.9	99.8 ± 0.6	98.9 ± 1.3	95.0 ± 3.2	98.5 ± 1.5
	(vi) smoothing, MSC, 2nd derivative	98.5 ± 1.5	94.7 ± 2.0	97.7 ± 1.8	98.3 ± 1.6	95.8 ± 2.7	98.5 ± 1.5
	(vii) smoothing, MSC, detrend	98.9 ± 1.5	92.8 ± 3.6	99.4 ± 1.0	98.6 ± 1.8	96.1 ± 3.6	98.5 ± 1.5

**Table 4:** Mean accuracy and precision of the prediction of the external test set for different pre-treatments and classification models for the differentiation of the black truffle species (29 *T. aestivum* samples, 10 *T. melanosporum* samples, and 11 *T. indicum* samples, all values in %).

			Classification Model				
		(a) LDA	(b) lin. SVM	(c) quad. SVM	(d) SSD	(e) RF	(f) *k*-NN
**pre-processing**	(i) MSC	98.7 ± 1.2	87.6 ± 1.2	90.4 ± 2.9	99.0 ± 1.0	90.3 ± 2.2	96.3 ± 2.1
	(ii) MSC, 1st derivative	98.9 ± 1.3	92.8 ± 2.2	94.9 ± 1.6	**99.1 ± 1.2**	91.7 ± 1.9	96.4 ± 1.8
	(iii) MSC, 2nd derivative	95.7 ± 2.2	79.5 ± 2.6	93.2 ± 2.3	93.7 ± 2.3	90.7 ± 2.5	90.0 ± 3.4
	(iv) MSC, detrend	98.7 ± 1.1	88.9 ± 1.4	90.5 ± 2.6	98.7 ± 1.1	91.8 ± 2.0	96.6 ± 2.2
	(v) smoothing, MSC, 1st derivative	98.9 ± 1.1	91.7 ± 2.1	95.4 ± 1.4	99.2 ± 1.1	92.0 ± 2.2	96.1 ± 1.9
	(vi) smoothing, MSC, 2nd derivative	98.8 ± 1.2	95.3 ± 2.0	98.8 ± 1.4	99.2 ± 1.1	92.7 ± 2.3	95.0 ± 2.7
	(vii) smoothing, MSC, detrend	98.8 ± 1.0	88.3 ± 1.4	90.4 ± 2.2	99.0 ± 1.0	91.9 ± 1.9	96.3 ± 2.0

**Table 5:** Confusion matrix for classification of the black truffle species with the build subspace discriminant model after MSC and 1st derivative; resulting in 99.1 ± 1.2% mean sensitivity. The predictions of 100 repetitions of the test set were accumulated.

		Predicted Species			
		*T. indicum*	*T. aestivum*	*T. melanosporum*	sensitivity [%]
**actual species**	*T. indicum*	1073	1	26	97.5
	*T. aestivum*	3	2897	0	99.9
	*T. melanosporum*	1	0	999	99.9
	specificity [%]	99.6	100	97.5	

**Table 6:** Mean accuracy and precision of the prediction of the external test set for different pre-treatments and classification models for the differentiation of Italian vs. non-Italian *T. magnatum* truffles (all values in %).

			Classification Model				
		(a) LDA	(b) lin. SVM	(c) quad. SVM	(d) SSD	(e) RF	(f) *k*-NN
**pre-processing**	(i) MSC	82.4 ± 4.5	51.8 ± 2.5	74.5 ± 5.0	82.6 ± 4.7	72.8 ± 5.4	80.2 ± 4.3
	(ii) MSC, 1st derivative	80.5 ± 4.5	50.8 ± 2.3	80.9 ± 3.4	82.0 ± 4.3	79.2 ± 5.6	82.5 ± 4.5
	(iii) MSC, 2nd derivative	83.6 ± 4.0	58.0 ± 3.6	82.9 ± 2.4	83.2 ± 3.1	88.4 ± 5.0	80.8 ± 4.5
	(iv) MSC, detrend	82.6 ± 4.7	51.7 ± 2.5	74.4 ± 4.9	81.3 ± 4.7	78.9 ± 6.6	81.6 ± 3.8
	(v) smoothing, MSC, 1st derivative	81.1 ± 4.1	51.4 ± 2.1	80.8 ± 3.0	81.1 ± 4.2	79.3 ± 5.4	80.9 ± 4.4
	(vi) smoothing, MSC, 2nd derivative	83.7 ± 4.2	63.8 ± 3.8	82.3 ± 3.6	82.5 ± 4.5	**82.8 ± 8.1**	81.8 ± 4.6
	(vii) smoothing, MSC, detrend	82.6 ± 5.0	51.4 ± 2.4	74.3 ± 4.9	81.8 ± 4.5	80.5 ± 6.6	81.5 ± 4.2

**Table 7:** Confusion matrix for classification for the differentiation of Italian vs. non-Italian T. magnatum truffles with the build RF model after smoothing. MSC and 2nd derivative; resulting in 82.8 ± 8.1% mean sensitivity. The predictions of 100 repetitions of the test set were accumulated.

		Predicted Origin		
		Italian	non-Italian	sensitivity [%]
**actual origin**	Italian	1247	153	89.1
	non-Italian	141	459	76.5
	specificity [%]	89.8	75.0	

## Supplementary Materials

The following figures and tables are available online at https://www.mdpi.com/2304-8158/9/7/922/s1, Figure S1: Influence of the order of pre-processing steps. (A) Raw data. (B) MSC and omitting the > 9000 cm^−1^ range. (C) Omitting the >9000 cm^−1^ range first and MSC; Table S1: Overview of the analysed truffle samples with number of samples, harvest year and country; Table S2: Mean accuracy and precision of the training set used for validation for different pre-treatment and classification models for the differentiation of the white truffle species (20 *T. magnatum* samples, 5 *T. borchii* samples, all values in %); Table S3: Mean accuracy and precision of the training set used for validation for different pre-treatment and classification models for the differentiation of the black truffle species (29 *T. aestivum* samples, 10 *T. melanosporum* samples and 11 *T. indicum* samples, all values in %); Table S4: Confusion matrix for classification of the white truffle species with the build linear SVM model after MSC and 1st derivative; resulting in 100% mean sensitivity. The predictions of 100 repetitions of the test set were accumulated; Table S5: Mean accuracy with standard deviation for different pre-treatment and classification models for the prediction of the test set for the differentiation of five truffle species (20 *T. magnatum* samples, 5 *T. borchii* samples, 29 *T. aestivum* samples, 10 *T. melanosporum* samples and 11 *T. indicum* samples, all values in %); Table S6: Mean accuracy and precision of the training set for different pre-treatment and classification models for the differentiation of the five truffle species (20 *T. magnatum* samples, 5 *T. borchii* samples, 29 *T. aestivum* samples, 10 *T. melanosporum* samples and 11 *T. indicum* samples, all values in %); Table S7: Confusion matrix for classification of five truffle species with the build subspace discriminant model after MSC and 1st derivative; resulting in 99.3 ± 0.9% mean sensitivity. The predictions of 100 repetitions of the test set were accumulated; Table S8: Mean accuracy and precision of the training set for different pre-treatment and classification models for the differentiation of Italian vs. non-Italian *T. magnatum* truffles (all values in %), MATLAB function for the creation of stratified parts for the nested cross validation.
