# Supplementary material for: Food Authentication: Truffle (Tuber spp.) Species Differentiation by FT-NIR and Chemometrics
Source: Foods. 2020 Jul 13;9(7):922. doi: 10.3390/foods9070922 (PMC7405009; doi:10.3390/foods9070922)
Supplement: Supplementary file 1 [file foods-09-00922-s001.zip › foods-853490-supplementary.pdf]

Supplementary Materials

Food authentication: Truffle (*Tuber* spp.) species differentiation by FT-NIR and chemometrics

Torben Segelke [1,‡], Stefanie Schelm [1,‡] Christian Ahlers [1] and Markus Fischer [1,*]

[1] HAMBURG SCHOOL OF FOOD SCIENCE - Institute of Food Chemistry, University of Hamburg, Grindelallee 117, 20146 Hamburg, Germany; torben.segelke@chemie.uni-hamburg.de (T.S.); stefanie.schelm@chemie.uni-hamburg.de (S.S.); christian.ahlers-2@studium.uni-hamburg.de (C.A.); Markus.Fischer@uni-hamburg.de (M.F.)

‡ Authors contributed equally

* Correspondence: Markus.Fischer@uni-hamburg.de; Tel.: +49-40-42838-4359

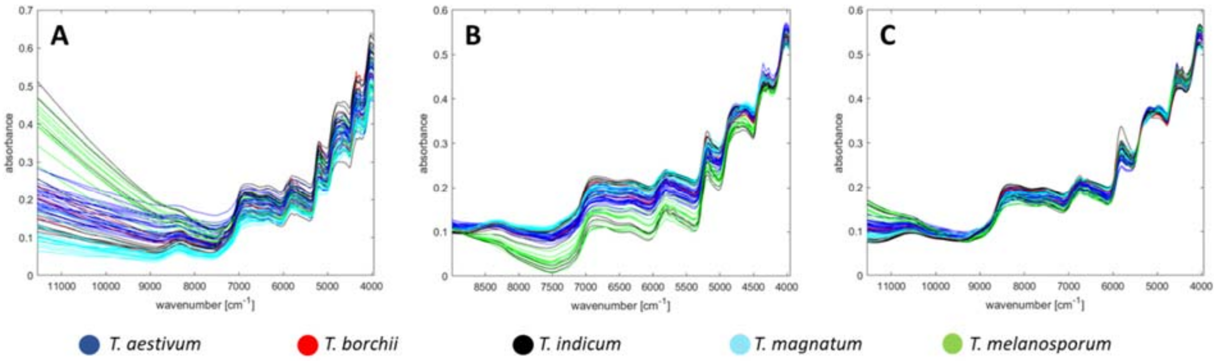

Figure S1: Influence of the order of pre-processing steps. (A) raw data. (B) MSC and omitting the > 9,000 cm^{-1} range. (C) omitting the > 9,000 cm^{-1} range first and MSC.

Table S1: Overview of the analysed truffle samples with number of samples, harvest year and country.

Tuber species	geographical origin	harvest year	number of samples
T. aestivum/	unkown	2018	7
T. uncinatum	Bulgaria	2019	2
	France	2019	1
	Iran	2019	1
	Italy	2018	3
	Italy	2019	1
	Italy, Tuscany, Florence	2018	1
	Moldova	2019	1
	Romania	2109	1
	Romania, Bihor, Oradea	2017	2
	Romania, Hunedoara, Deva	2017	1
	Romania, Moldau, Bacau	2017	4
	Romania, Salaj, Zalau	2017	1
	Romania, South-Italy	2017	1
	Romania, Timisoara	2019	1
	Slovenia	2019	1
			sum: 29
T. albidum	Italy	2018	1
	Italy	2019	1
	Italy, Basilicata	2020	1
	Italy, Emilia-Romagna, Faenza	2019	1
	Spain	2019	1
			sum: 5
T. indicum	China	2017	1
	China	2018	3
	China	2019	4
	China	2020	2
	China, Yunnan, Dali City	2019	1
			sum: 11
T. magnatum	Bulgaria	2019	1
	Bulgaria, Sliven, Sliven	2018	1
	Croatia	2019	1
	Croatia, Istria, Buzet	2018	1
	Italy	2018	3
	Italy, Abruzzo, L'Aquila	2018	1
	Italy, Campania, Naples	2018	1
	Italy, Lazio, Rome	2018	1
	Italy, Marche, Ancona	2018	1
	Italy, Molise, Campobasso	2018	1
	Italy, Piedmont	2019	1
	Italy, Piedmont, Turin	2018	1
	Italy, Romagna	2018	1
	Italy, Savigno	2019	1

Table S1 continued

Tuber species	geographical origin	harvest year	number of samples
T. magnatum	Italy, Tuscany	2019	1
	Italy, Umbria, Perugia	2018	1
	Romania	2019	1
	Romania, Targu Jiu, Targu Jiu	2018	1
			sum: 20
T. melanosporum	Australia	2018	1
	Australia	2019	1
	France	2018	1
	France, Languedoc, Laudun-l'Ardoise	2019	1
	Italy, Abruzzo	2020	1
	Italy, Marche	2018	1
	Spain, Teruel, Sarrion	2019	1
	Spain, Valencia, Castellon	2019	3
			sum: 10

Table S2: Mean accuracy and precision of the training set used for validation for different pre-treatment and classification models for the differentiation of the white truffle species (20 *T. magnatum* samples, 5 *T. borchii* samples, all values in %).

		classification model					
		a) LDA	b) lin. SVM	c) quad. SVM	d) SSD	e) RF	f) *k*-NN
**pre-processing**	i) MSC	96.2 ± 3.7	91.6 ± 2.6	90.7 ± 3.5	94.0 ± 3.2	86.2 ± 4.6	97.4 ± 2.6
	ii) MSC, 1st derivative	99.3 ± 1.5	93.0 ± 2.6	96.1 ± 3.1	94.4 ± 3.7	89.2 ± 2.9	96.1 ± 2.1
	iii) MSC, 2nd derivative	95.8 ± 2.1	98.3 ± 2.4	98.0 ± 2.5	98.8 ± 1.6	80.7 ± 7.9	96.3 ± 2.2
	iv) MSC, detrend	95.8 ± 3.4	85.6 ± 4.0	92.0 ± 2.6	94.0 ± 2.2	79.1 ± 7	88.1 ± 3.2
	v) smoothing, MSC, 1st derivative	99.1 ± 1.6	93.6 ± 3.1	97.3 ± 2.6	94.7 ± 3.5	85.7 ± 3	95.8 ± 2.8
	vi) smoothing, MSC, 2nd derivative	96.1 ± 2.4	93.9 ± 2.8	96.2 ± 2.7	94.3 ± 3.4	86.3 ± 3.8	95.8 ± 2.9
	vii) smoothing, MSC, detrend	96.0 ± 3.8	98.6 ± 2.2	97.8 ± 2.5	98.1 ± 1.9	79.8 ± 7.1	96.1 ± 2.1

Table S3: Mean accuracy and precision of the training set used for validation for different pre-treatment and classification models for the differentiation of the black truffle species (29 *T. aestivum* samples, 10 *T. melanosporum* samples and 11 *T. indicum* samples, all values in %).

		classification model					
		a) LDA	b) lin. SVM	c) quad. SVM	d) SSD	e) RF	f) *k*-NN
**pre-processing**	i) MSC	94.4 ± 2.9	92.0 ± 2.2	89.8 ± 1.9	97.4 ± 1.2	84.0 ± 4.5	87.6 ± 1.9
	ii) MSC, 1st derivative	96.0 ± 1.5	88.4 ± 4.5	86.8 ± 2.1	95.8 ± 1.7	77.6 ± 4.7	85.9 ± 4.3
	iii) MSC, 2nd derivative	83.4 ± 4.4	88.3 ± 5.0	91.3 ± 2.4	95.5 ± 1.3	78.6 ± 6.0	85.6 ± 3.6
	iv) MSC, detrend	95.0 ± 3.2	60.2 ± 4.2	72.3 ± 4.4	77.3 ± 3.6	67.7 ± 9.2	58.9 ± 4.5
	v) smoothing, MSC, 1st derivative	96.0 ± 1.5	88.5 ± 3.9	86.9 ± 2.3	95.9 ± 1.8	82.8 ± 3.7	85.9 ± 3.8
	vi) smoothing, MSC, 2nd derivative	95.9 ± 2.8	89.8 ± 3.8	86.6 ± 2.3	96.1 ± 1.8	81.7 ± 3.6	86.3 ± 3.5
	vii) smoothing, MSC, detrend	94.8 ± 2.5	90.0 ± 4.3	91.5 ± 2.2	95.9 ± 1.2	78.7 ± 7.2	86.2 ± 3.1

Table S4: Confusion matrix for classification of the white truffle species with the build linear SVM model after MSC and 1st derivative; resulting in 100% mean sensitivity. The predictions of 100 repetitions of the test set were accumulated.

		predicted species		
		T. magnatum	*T. borchii*	sensitivity [%]
**actual species**	*T. magnatum*	2000		100
	T. borchii		500	100
	specificity [%]	100	100	100

Table S5: Mean accuracy with standard deviation for different pre-treatment and classification models for the prediction of the test set for the differentiation of five truffle species (20 *T. magnatum* samples, 5 *T. borchii* samples, 29 *T. aestivum* samples, 10 *T. melanosporum* samples and 11 *T. indicum* samples, all values in %).

		classification model					
		a) LDA	b) lin. SVM	c) quad. SVM	d) SSD	e) RF	f) *k*-NN
**pre-processing**	i) MSC	99.2 ± 0.8	82.8 ± 1.3	90.7 ± 2.0	99.3 ± 1.0	87.9 ± 2.3	95.9 ± 1.5
	ii) MSC, 1st derivative	99.2 ± 0.8	85.3 ± 1.4	94.3 ± 1.2	**99.3 ± 0.9**	86.9 ± 2.2	96.1 ± 1.5
	iii) MSC, 2nd derivative	94.5 ± 1.9	75.0 ± 2.0	91.0 ± 1.8	93.9 ± 1.9	79.6 ± 2.7	89.3 ± 2.5
	iv) MSC, detrend	99.2 ± 0.7	83.1 ± 1.3	88.1 ± 2.2	99.2 ± 1.1	88.2 ± 2.3	96.4 ± 1.7
	v) smoothing, MSC, 1st derivative	99.3 ± 0.9	86.2 ± 3.4	95.0 ± 3.5	99.2 ± 3.8	87.3 ± 3.8	96.0 ± 2.5
	vi) smoothing, MSC, 2nd derivative	95.9 ± 1.8	88.6 ± 1.9	93.7 ± 1.7	98.2 ± 1.2	86.4 ± 3.6	92.8 ± 2
	vii) smoothing, MSC, detrend	99.3 ± 0.7	83.0 ± 1.2	88.6 ± 1.9	99.3 ± 1.1	88.9 ± 2.4	95.8 ± 1.8

Table S6: Mean accuracy and precision of the training set for different pre-treatment and classification models for the differentiation of the five truffle species (20 *T. magnatum* samples, 5 *T. borchii* samples, 29 *T. aestivum* samples, 10 *T. melanosporum* samples and 11 *T. indicum* samples, all values in %).

		classification model					
		a) LDA	b) lin. SVM	c) quad. SVM	d) SSD	e) RF	f) *k*-NN
**pre-processing**	i) MSC	97.1 ± 1.7	83.9 ± 2.4	86.7 ± 2.6	97.1 ± 0.9	80.5 ± 4.4	87.1 ± 2.5
	ii) MSC, 1st derivative	96.6 ± 1.7	86.5 ± 2	86.2 ± 2.2	97 ± 1	71.1 ± 5	87 ± 3.1
	iii) MSC, 2nd derivative	80.8 ± 3.5	86.6 ± 2.4	89 ± 1.8	96.2 ± 1.1	67.6 ± 6.1	87.6 ± 3.1
	iv) MSC, detrend	97.1 ± 1.6	62.3 ± 3.6	71.8 ± 3.1	76.8 ± 3.2	67.3 ± 6.8	56.8 ± 4.1
	v) smoothing, MSC, 1st derivative	97.1 ± 1.4	87.4 ± 2.3	85.4 ± 2.4	97.1 ± 1.1	71.7 ± 3.4	86 ± 3.1
	vi) smoothing, MSC, 2nd derivative	86.3 ± 4.1	86.9 ± 2.4	85 ± 2.3	96.9 ± 1.2	72 ± 3.3	86.5 ± 3
	vii) smoothing, MSC, detrend	96.9 ± 1.5	87.1 ± 2.5	89.5 ± 1.5	96.8 ± 1	70.3 ± 5.1	86.9 ± 2.3

Table S7: Confusion matrix for classification of five truffle species with the build subspace discriminant model after MSC and 1st derivative; resulting in 99.3 ± 0.9% mean sensitivity. The predictions of 100 repetitions of the test set were accumulated.

		predicted species					
		T. indicum	*T. aestivum*	*T. magnatum*	*T. melanosporum*	*T. borchii*	sensitivity [%]
**actual species**	*T. indicum*	1077	0	0	23	0	97.9
	T. aestivum	0	2900	0	0	0	100
	T. magnatum	0	22	1978	0	0	98.9
	T. melanosporum	1	0	0	999	0	99.9
	T. borchii	0	0	0	0	500	100
	specificity [%]	99.9	99.2	100	97.7	100	

Table S8: Mean accuracy and precision of the training set for different pre-treatment and classification models for the differentiation of Italian vs. non-Italian *T. magnatum* truffles (all values in %).

		**classification model**					
		a) LDA	b) lin. SVM	c) quad. SVM	d) SSD	e) RF	f) *k*-NN
**pre-processing**	i) MSC	48.3 ± 14	65.4 ± 4.5	50.0 ± 8.8	50.3 ± 9.9	47.6 ± 10.3	44.6 ± 7.8
	ii) MSC, 1st derivative	51.5 ± 8.5	67 ± 4.4	49.2 ± 9.8	47.4 ± 10.5	40.6 ± 11.5	46.8 ± 8.5
	iii) MSC, 2nd derivative	63.7 ± 6.8	71.7 ± 2.6	57.3 ± 6.5	56.5 ± 4.8	52.5 ± 15	50.5 ± 9.6
	iv) MSC, detrend	46.9 ± 12	72.7 ± 2.3	65.2 ± 5.6	67.2 ± 4.8	61.3 ± 17.5	51.4 ± 11.3
	v) smoothing, MSC, 1st derivative	50.3 ± 8.9	65.3 ± 4.7	48.2 ± 10.2	46.5 ± 8.7	49.7 ± 13.2	50.2 ± 8.7
	vi) smoothing, MSC, 2nd derivative	51.8 ± 9.9	65.2 ± 5.9	47.1 ± 10.1	47.6 ± 9.8	49.4 ± 12	49.8 ± 9.0
	vii) smoothing, MSC, detrend	45.8 ± 13	70.8 ± 3.4	55.2 ± 6.3	54.4 ± 6.4	47.2 ± 13.4	45.8 ± 9.2

MATLAB function for the creation of stratified parts for the nested cross validation:

```
function [y,z] = create_strat_parts(x,c,k)
% function gives back stratified parts for a nested cross validation in y
with their respective categories in z.
% function divides matrix x with its categories in c in k parts
stratifiedly distributed
%x: matrix with values which is ought to split into stratified parts
%c: numerical categories of the samples in x
%k: number of stratified parts to create

% written by Torben Segelke, Hamburg School of Food Science, 200405,200411

%Schritt1: Bereiche festlegen, neue Matrizen festlegen
sz=size(x); %sz ist ein 1x2-vector mit der Anzahl an Zeilen mal Spalten
rest=mod(sz(1,1),k);
anz=(sz(1,1)-rest)/k; %in jedem der k Parts sind mindestens anz Proben

h=zeros(max(c),2); %für den categorialen Vektor c ermitteln, wie viele
Proben jeweils pro Kategorie vorkommen und dies in Matrix h speichern, dann
über zwei for schleifen füllen
for i=1:max(c)
    h(i,1)=i;
end
for i=1:length(c)
    h(c(i,1),2)=h(c(i,1),2)+1;
end

%die Matrix a aufdröseln je nach Kategorie und in neuer matrix eintragen,
%dafür erst Bereich festlegen, dann füllen
for i=1:max(c)
    xneu{i}=zeros(h(i,2),sz(1,2));
    counter{i}=ones(1,1);
end
for i=1:length(c)
    xneu{c(i,1)}(counter{c(i,1)},:)=x(i,:);
    counter{c(i,1)}(1,1)=counter{c(i,1)}(1,1)+1;
end

%Schritt2: leere Parts erstellen
if rest==0
    for i=1:k
        y{i}=zeros(anz,sz(1,2));
        z{i}=zeros(anz,1);
    end
else
    for i=1:rest %i=k-rest+1:k
        y{i}=zeros(anz+1,sz(1,2));
        z{i}=zeros(anz+1,1);
    end
    for i=rest+1:k %i=1:k-rest
        y{i}=zeros(anz,sz(1,2));
        z{i}=zeros(anz,1);
    end

end

%Schritt3: Parts erstellen, Zufallsvektoren definieren
for i=1:max(c)
    zfll{i}=1:h(i,2);
```

```
    zfll{i}=zfll{i}'; %invertieren
    zfll{i}=zfll{i}(randperm(length(zfll{i}))); %zufallsdurchmischen

end

for i=1:k %wie bereits oben schonmal eine ZählVariable einführen, damit das
Programm in den Folge-Schleifen weiß, wo die nächste Variable
hingeschrieben werden soll
countP{i}=ones(1,1);
end
for i=1:max(c) %wie bereits oben schonmal eine ZählVariable einführen, ruft
hier den zufallsvektor nacheinander ab
countz{i}=ones(1,1);
end
countk=1; %um die Nachzügler zu verteilen

%hier beginnt die eigentliche Schleife zum Verteilen
for i=1:max(c)
    if mod(h(i,2),k)==0 %wenn diese Abfrage stimmt, liegen für eine
Kategorie n*k Proben vor, sodass perfekt verteilt werden kann
        n=h(i,2)/k; %wie viele n liegen vor?
            for nn=1:n
                for j=1:k
                    y{j}(countP{j}(1,1),:)=xneu{i}(zfll{i}(countz{i},1),:);
                    z{j}(countP{j}(1,1),1)=i;
                    countz{i}(1,1)=countz{i}(1,1)+1;
                    countP{j}(1,1)=countP{j}(1,1)+1;
                end
            end
    else %hier geht es nicht perfekt auf
        n=(h(i,2)-mod(h(i,2),k))/k; %wie viele Runden können normal
durchgeführt werden?
            for nn=1:n
                for j=1:k
                    y{j}(countP{j}(1,1),:)=xneu{i}(zfll{i}(countz{i},1),:);
                    z{j}(countP{j}(1,1),1)=i;
                    countz{i}(1,1)=countz{i}(1,1)+1;
                    countP{j}(1,1)=countP{j}(1,1)+1;
                end
            end
        %weitere Runden für die Nachzügler:
        nnach=h(i,2)-n*k; %wie viele Proben noch zuverteilen
            for nn=1:nnach

y{countk}(countP{countk}(1,1),:)=xneu{i}(zfll{i}(countz{i},1),:);
                    z{countk}(countP{countk}(1,1),1)=i;
                    countz{i}(1,1)=countz{i}(1,1)+1;
                    countP{countk}(1,1)=countP{countk}(1,1)+1;
                    if countk==k %um countk ggf zu resetten
                        countk=1;
                    else
                        countk=countk+1;
                    end
            end

    end


end
```
